# Cell cycle and apoptosis regulation by NFAT transcription factors: new roles for an old player

**DOI:** 10.1038/cddis.2016.97

**Published:** 2016-04-21

**Authors:** G P Mognol, F R G Carneiro, B K Robbs, D V Faget, J P B Viola

**Affiliations:** 1 Programa de Biologia Celular, Instituto Nacional de Câncer, Rio de Janeiro, Brazil; 2 Department of Signaling and Gene Expression, La Jolla Institute for Allergy and Immunology, La Jolla, CA, USA; 3 Laboratory for Proteomics and Protein Engineering, Carlos Chagas Institute, FIOCRUZ-Paraná, Brazil; 4 Department of Basic Sciences (FCB), Universidade Federal Fluminense, Nova Friburgo, Brazil

## Abstract

The NFAT (nuclear factor of activated T cells) family of transcription factors consists of four Ca^2+^-regulated members (NFAT1–NFAT4), which were first described in T lymphocytes. In addition to their well-documented role in T lymphocytes, where they control gene expression during cell activation and differentiation, NFAT proteins are also expressed in a wide range of cells and tissue types and regulate genes involved in cell cycle, apoptosis, angiogenesis and metastasis. The NFAT proteins share a highly conserved DNA-binding domain (DBD), which allows all NFAT members to bind to the same DNA sequence in enhancers or promoter regions. The same DNA-binding specificity suggests redundant roles for the NFAT proteins, which is true during the regulation of some genes such as *IL-2* and *p21*. However, it has become increasingly clear that different NFAT proteins and even isoforms can have unique functions. In this review, we address the possible reasons for these distinct roles, particularly regarding N- and C-terminal transactivation regions (TADs) and the partner proteins that interact with these TADs. We also discuss the genes regulated by NFAT during cell cycle regulation and apoptosis and the role of NFAT during tumorigenesis.

## Facts

- NFAT proteins regulate cell cycle-, apoptosis-, angiogenesis- and metastasis-related genes;
- Although they bind to the same DNA sequence, different NFAT members and even isoforms have both redundant and opposite functions;
- NFAT proteins have a weak DNA-binding capacity and frequently cooperate with other transcriptional partners;
- NFAT signaling and expression are deregulated in cancer.

## Open Questions

- The binding of partner proteins to the less conserved transactivation region (TAD) domains of different NFAT members and isoforms might be responsible for the non-redundant functions of NFAT. What are these proteins?
- T-cell activation is an example where the presence/absence of a specific partner, AP-1, dictates the program of gene expression driven by NFAT, determining the mode of immune response. It remains to be shown whether other partners are also pivotal for the functions mediated by NFAT proteins in specific contexts or cell types.
- NFAT proteins are relevant factors in tumorigenesis and tumor progression. What are the subsets of genes regulated by NFAT in the different stages of tumor progression? There are specific NFAT isoforms expressed in tumor samples? Is it possible to modulate NFAT activity in order to get therapeutic response in cancer?

The NFAT (nuclear factor of activated T cells) family of transcription factors consists of five members (NFAT1–NFAT5) encoded by different genes, first described in T lymphocytes as an inducible nuclear factor that could bind and transactivate the IL-2 promoter.^1^ Currently, it is clear that these proteins are also expressed in other immune cells^2, 3^ and non-immune cells, such as cartilage cells,^4^ adipocytes,^5^ pancreatic,^6^ breast^7^ and cardiac cells.^8^ In addition to their well-documented role in T lymphocytes, where they control gene expression during cell activation and differentiation,^9, 10^ NFAT also regulates genes involved in the cell cycle, apoptosis, angiogenesis and metastasis.^7, 10, 11, 12^

**Figure 1:**
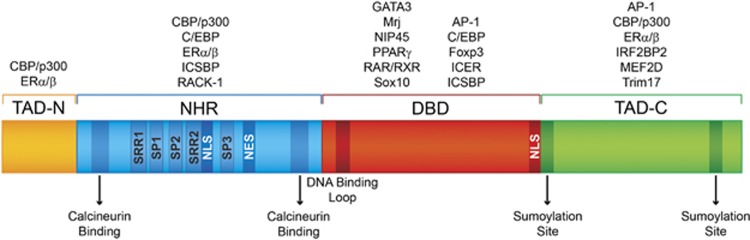
Schematic primary structure of NFAT. The calcium-regulated NFAT proteins are composed of the NHR, the DBD and the N- and C-terminus transactivation domains (TAD-N and TAD-C, respectively). The NHR displays two calcineurin-binding sites, a nuclear localization sequence (NLS) and a nuclear export signal (NES). Another NLS is located in the DBD region. The NFAT serine residues that are dephosphorylated upon calcineurin activation are located in the SRR1, SRR2, SP1, SP2 and SP3 regions. The NFAT DBD region that directly contacts DNA is indicated as the DNA-binding loop. The TAD-C region of NFAT1 and NFAT2 has two sumoylation sites. Different NFAT partner proteins interact with specific domains of NFAT, and these partners are listed above each NFAT domain

Besides NFAT5, which is activated in response to osmotic stress,^13^ all the other NFAT proteins (NFAT1–4) are regulated by the calcium and calcineurin signaling pathways.^14, 15^ The conserved regions of calcium-regulated NFAT proteins consist of two tandem domains: (1) the regulatory domain, which is also known as the NFAT-homology region (NHR) and (2) the DNA-binding domain (DBD), also known as the Rel-homology region. The NHR is moderately conserved, sharing 22–36% sequence identity among the different NFAT members (Figure 1). The DBD comprises ~270 amino acids and shares 64–72% sequence identity among the different NFAT members (Figure 1). This highly conserved domain confers the specificity to bind the DNA core sequence (A/T)GGAAA^14^ to all the NFAT proteins. Flanking the NHR and the DBD domains are two transcriptional activation domains (TAD) at the N- and C termini (Figure 1), which are highly variable among the different NFAT members and isoforms.^14, 15^

**Figure 2:**
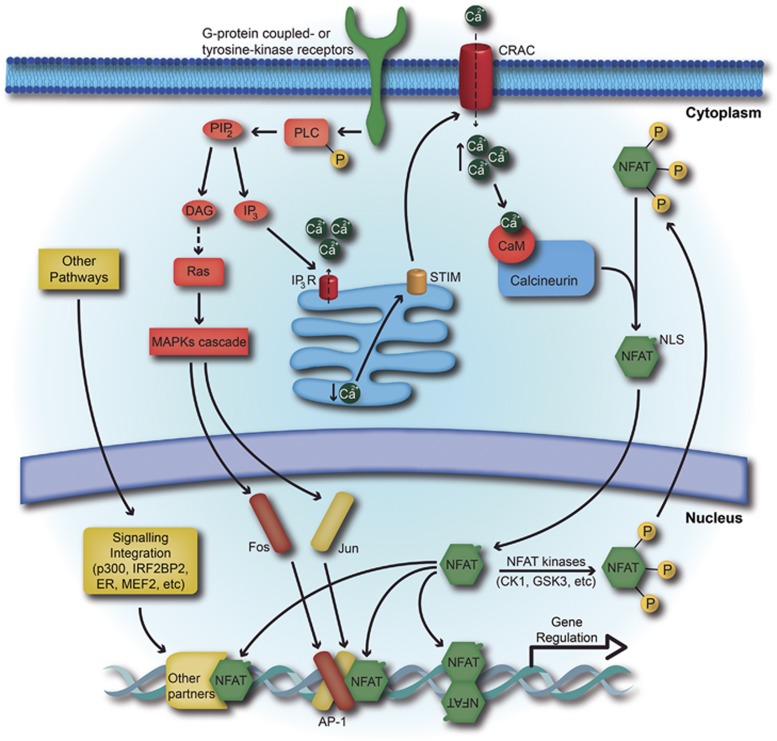
The NFAT signaling pathway. Representation of NFAT activation and translocation to the nucleus. The triggering of a G-protein-coupled or tyrosine-kinase receptor leads to the activation of phospholipase C, which promotes the hydrolysis of phosphatidylinositol-4,5-biphosphate (PIP_2_), producing diacylglycerol and inositol-1,4,5-triphosphate (IP_3_). Then, IP_3_ binds to IP_3_R and induces the release of Ca^2+^ ions from the endoplasmic reticulum to the cytoplasm, depleting the intracellular stores. The depletion of calcium stores induces the activation of the stromal interaction molecule, which promotes the opening of calcium release-activated channel in the plasma, leading to the sustained increase in the cytoplasmic calcium levels. Next, the Ca^2+^ ions bind to calmodulin and activate the phosphatase calcineurin. Calcineurin dephosphorylates specific serine residues in NFAT. Dephosphorylated NFAT undergoes a conformational change exposing its NLS and translocates to the nucleus. In the nucleus, NFAT binds to DNA alone or in cooperation with other nuclear partner proteins, activated by other signaling pathways, to regulate gene expression. To reverse its activation, NFAT kinases rephosphorylate NFAT and promote the concealment of its NLS and exposure of the NES, redirecting NFAT to the cytoplasm

The NHR is highly phosphorylated in resting cells, keeping NFAT in an inactive state and restricted to the cytoplasm (Figure 2). An increase in intracellular calcium levels activates calcineurin, a calcium/calmodulin-dependent serine/threonine phosphatase, which dephosphorylates the NHR, exposing the NFAT1–4 nuclear localization signal and inducing NFAT translocation to the nucleus^14, 16, 17^ (Figure 2). Once in the nucleus, NFAT proteins can bind to their target promoter elements and regulate the transcription of specific responsive genes, either alone or in combination with other nuclear partner proteins^9^ (Figure 2).

Although NFAT proteins bind to the same DNA sequence, these proteins have both redundant and non-redundant roles during the regulation of different processes. In this review, we discuss the non-conserved TAD regions that could explain at least in part this dual role, together with the NFAT partners that interact with the variable TADs. In addition, we highlight the regulation of cell cycle and apoptosis genes by the different NFAT proteins and the function of NFAT in malignant transformation and tumorigenesis.

## TAD-conserved Regions

**Figure 3:**
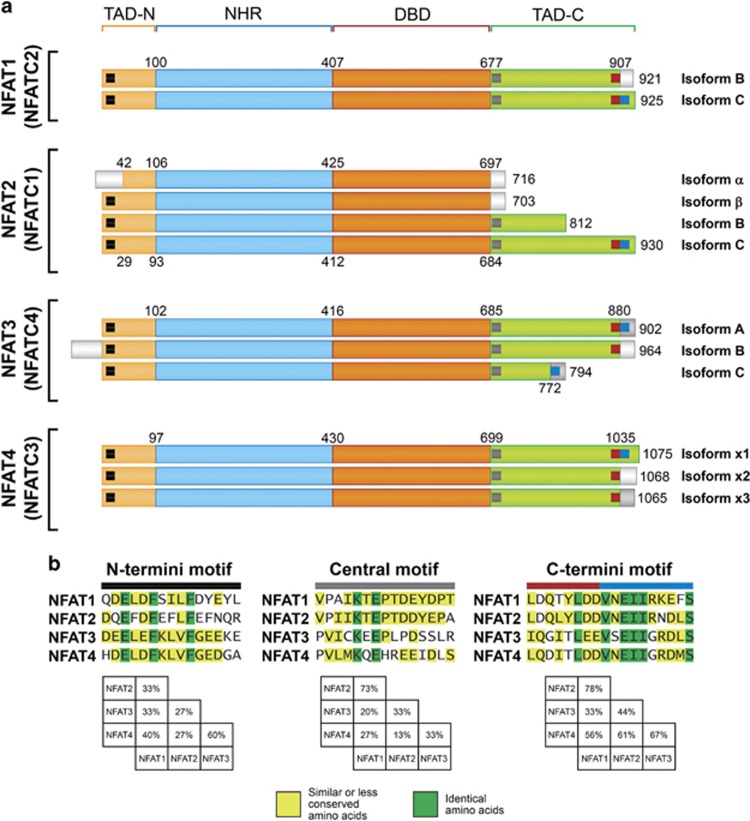
The NFAT family of transcription factors. (**a**) Schematic primary structure of human calcium-regulated NFAT proteins and their isoforms. The alternative nomenclature for the NFAT genes is shown in parenthesis. As in Figure 1, NHR, DBD and TADs are represented. In addition, the TAD-conserved regions are indicated by a black square (N-terminus motif); a gray square (central motif); and by red and blue squares (C-terminus motif). The NCBI reference numbers for the NFAT isoforms are: NP_036472.2 (NFAT1-B); NP_775114.1 (NFAT1-C); NP_765978.1 (NFAT2-*α*); NP_001265604.1 (NFAT2-*β*); NP_765977.1 (NFAT2-B); NP_765975.1 (NFAT2-C); NP_004545.2 (NFAT3-A); NP_001129494.1 (NFAT3-B); NP_001185894.1 (NFAT3-C); NP_775188.1 (NFAT4-x1); NP_004546.1 (NFAT4-x2); NP_775186.1 (NFAT4-x3). (**b**) Alignment of the NFAT TAD-conserved regions, designated as the N-terminus motif, central motif and C-terminus motif. Amino acids marked with yellow indicate that residues are similar or occur more than 50% of the time at that position; green indicates identical residues. The similarity percentage for each region between the NFAT proteins is also shown

The TADs are putative sites where distinct NFAT proteins interact with specific partners and control important biological functions such as proliferation and cell death. They are the least conserved domains of NFAT. However, even these regions have some degree of conservation (Figure 3). Three main small conserved regions can be identified among the TADs of NFAT variants and will be referred to as the N-terminus motif (black square, Figure 3), central motif (gray square) and C-terminus motif (sub-motif 1, red square; sub-motif 2, blue square).

The N-terminus motif is present in NFAT variants depending on the initial exon used for transcription. The NFAT2-*α* autoregulatory loop is one mechanism where the N-terminus motif is excluded. In a resting state, the transcription initiation of NFAT2 is in exon 2, which encodes the N-terminus motif. After activation, NFAT binds to the NFAT2 promoter, leading to initiation of transcription from exon 1 and skipping exon 2.^18^ The N-terminus motif is rich in acidic and hydrophobic residues (Figure 3b), and it can act as an acidic activation domain (AAD).^14, 19^ The AADs are among the most potent transcriptional activators described.^20^ In fact, it has been shown that the NFAT2-*β* amino-acid region from 1 to 30 (region containing the N-terminus motif, Figure 3a) is necessary and sufficient to elicit high *in vitro* transcription.^19^ Furthermore, the NFAT1-C amino-acid region 1–144 also contains a potent transactivation domain.^21^ Moreover, the importance of this motif is not restricted to transcriptional activation. It was shown that casein kinase 1 specifically binds to the N-terminus motif and induces NHR phosphorylation and nuclear export of NFAT,^22^ with consequent NFAT inactivation. Because several NFAT isoforms lack the N-terminus motif (Figure 3a), this splicing variation might facilitate the nuclear localization and function of NFAT.

The NFAT small central motif is located immediately after the DBD and is composed of a highly conserved region of 15 amino acids present in most of NFAT family members besides NFAT2-*α* and *β* (gray square; Figure 3). Apparently, the central motif is not involved in transcriptional activation.^23^ However, it was shown that the sumoylation of the well-conserved lysine residue (K; Figure 3b) in the central motif of both NFAT1 and NFAT2 is tightly linked to NFAT nuclear localization and transcriptional function.^24, 25^ Therefore, the presence of this small central motif might be important for the function mediated by different NFATs.

The C-terminus motif is located in the last 50 amino acids of C-terminus transactivation domain (TAD-C; Figure 3b) and appears to be important for transactivation.^21, 23^ This motif has a sequence identity of ~50% among different NFATs and can be divided into two sub-motifs that are present in different exons and are subjected to splicing variation: sub-motif 1 and 2 (red and blue squares, respectively; Figure 3). NFAT1-C contains both sub-motifs and induces CAT expression at least two times greater than NFAT1-B, which only has the first sub-motif.^21^ The same phenotype was observed for NFAT4 isoforms that lack sub-motif 2.^26^ Although several NFAT splicing variants have been established, as reviewed by Vihma *et al.*,^27^ little is known about the functional meaning of these different proteins; however, it is reasonable to expect that they exert specific functions depending on the conserved regions present in each isoform.

## NFAT Partners

NFAT proteins can interact with several transcription factors and regulators in the nucleus, being important integrators of calcium signals with other signaling pathways.^15, 28^ The interaction of NFAT with other proteins through their conserved DBD and NHR regions can result in synergistic activation^29, 30, 31, 32, 33, 34, 35, 36, 37, 38, 39, 40, 41, 42, 43, 44, 45, 46^ (such as with GATA-3;^38^ ICSBP;^42^ C/EBP;^43^ AP-1^41, 44^) or repression^47, 48, 49, 50, 51, 52, 53^ (PPARγ^47, 48^ Mrj;^49^ Foxp3;^50, 51^ ICER^52^) of NFAT-mediated transcription (Table 1). Although the interactions with DBD and NHR regions are important for NFAT function, in this section, we will focus on the NFAT partners that interact with the less conserved NFAT TAD regions.

Besides the conserved motifs in the TAD regions discussed in the previous section, both the amino- and carboxyl-terminal ends of the NFAT family proteins show low sequence conservation. It is possible that different NFAT TADs could account for the non-redundant functions of these transcription factors by distinct control of transcription or through cooperation with the different protein partners, such as described below:

### CBP/p300

CBP and p300 directly interact with amino acids 1–145 of NFAT1 and 1–108 of NFAT2.^54, 55^ NFAT2 further interacts with CBP/p300 at the NHR amino acids 113–205.^55^ CBP also interacts with the NHR (amino acids 130–237) and with the TAD-C (amino acids 771–902) regions of the NFAT3 protein.^56^ Both NFAT3 regions seem to be fundamental for CBP binding.^56^ Because NFAT TAD is a highly variable region of NFAT proteins (Figure 3a), it would be of interest to analyze CBP/p300 interactions with different NFAT splice variants. Once CBP/p300 interacts with multiple NFAT regions, a differential transcriptional activity may be achieved by various NFAT splicing isoforms.

### ER*α*/ER*β*

Both ER*α* and ER*β* interact with NFAT3-A through residues 1–261 (TAD-N (N-terminus transactivation domain)/NHR), 261–450 (NHR) and 613–902 (TAD-C).^57, 58^ The NFAT3–ER complex dramatically reduced the transactivation of the IL-2 promoter, suggesting that ER can function as an NFAT co-repressor.^58^ Interestingly, NFAT3 can act in synergy with ER to transactivate elements containing ER- but not NFAT-binding sites, suggesting cross-talk between NFAT and ER: when the NFAT3–ER complex is bound to a NFAT element, it functions as a repressor of transcription, and, once bound to the ER element, as an activator.^57^

### MEF2

The transcription factor MEF2 interacts with NFAT1-C TAD-C (residues 679–927)^59^ but not with NFAT2-*α* or NFAT3-A.^60^ Because NFAT2-C and NFAT1-C TAD-C regions exhibit some conservation (Figure 3), it would be interesting to verify whether MEF2 can interact with the NFAT2-C protein as well. The MEF2–NFAT1 interaction leads to synergistic activation of pathways involved in cell death, muscle development,^60, 61^ thymocyte-negative selection and apoptosis.^59, 60^

### IRF2BP2

IRF2BP2 interacts with the C-terminal region of NFAT1 and strongly inhibits its transcriptional activity during the regulation of cytokine genes.^62^ Interestingly, no interaction was detected between IRF2BP2 and the other NFAT family members, indicating that IRF2BP2 is a NFAT1-specific partner and could be responsible for some repression functions mediated by NFAT1 but not by the other NFAT members.

### Trim17

It was shown that Trim17 interacts with the C-terminal end of both NFAT3 and NFAT4 in neuronal cells.^63^ In addition, sumoylated sites of NFAT4 but not NFAT3 are necessary for the interaction. Trim17 inhibits the activity of NFAT3–4 by favoring their cytoplasmic localization. Thus, this interaction shows a new mechanism of NFAT3–4 regulation. The effect of Trim17 on the NFAT1–2 cellular localization was not investigated.

The interaction between all of these proteins and the TAD regions of distinct NFAT members can strongly contribute to our knowledge of the non-redundant phenotypes observed both in mice lacking individual NFAT proteins and in many *in vitro* studies as described below.

## Cell Cycle and Apoptosis Regulation by NFAT

The specific role of each NFAT member in the control of gene transcription during cell cycle and apoptosis is not completely clear, mainly because most of the studies did not evaluate different NFAT members in the same model. We clearly demonstrated that ectopic expression of constitutively active forms of NFAT proteins (CA-NFAT1-C and CA-NFAT2-*α*) produces opposite phenotypes in the regulation of the cell cycle and apoptosis in NIH3T3 cells. Whereas NFAT2 acts as a positive regulator of cell proliferation and a repressor of cell death, NFAT1 induces a slight cell cycle arrest and a significant increase in cell death.^64^ A truncated CA-NFAT1 protein that lacks the TAD-C domain was unable to induce apoptosis, showing that the C-terminal domain of NFAT1 is responsible for its pro-apoptotic characteristic.^64^ More recently, it became clear that not just the NFAT isoform, but also the cell type and the signaling pathways activated contribute for the distinct functions exerted by the NFAT proteins. Below, we discuss the role of NFAT proteins during the regulation of genes involved in the control of the cell cycle and apoptosis.

## NFAT and Cell Cycle Regulation

**Figure 4:**
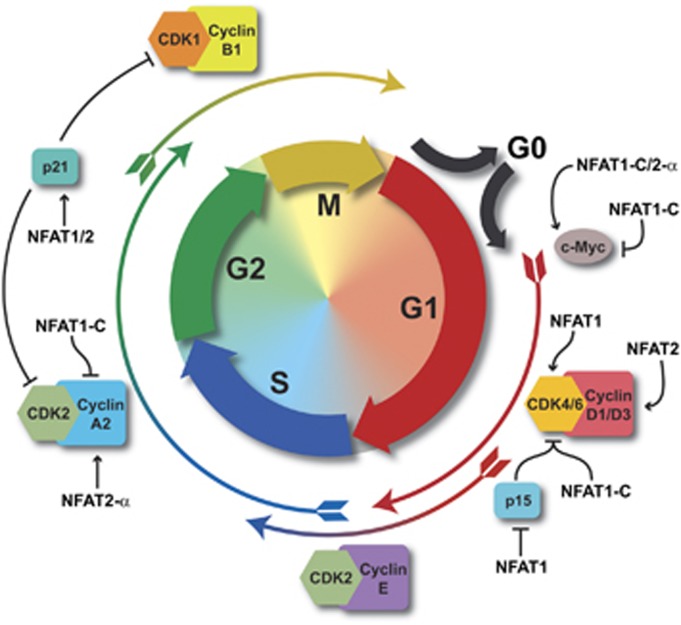
Cell cycle regulation by NFAT proteins. Schematic representation of the cell cycle regulation. The cell cycle phases G1, S, G2 and M are indicated. Non-dividing cells are in G0. The specific complexes of Cyclin/Cyclin-dependent kinases (CDK) and the CDK inhibitors p15 and p21 are indicated in specific cell cycle phases. The proto-oncogene c-Myc is also shown. NFAT proteins control cell cycle progression by regulating the expression of some cell cycle genes. The negative or/and positive regulations driven by NFAT proteins on these cell cycle-related genes are indicated

Evidence that NFAT proteins are involved in cell cycle control emerged from observations that DNA synthesis due to stimulation with growth factors is Ca^2+^-dependent and is markedly inhibited by CsA and FK506,^65^ drugs that inhibit calcineurin activity and, consequently, NFAT activation.^14^ Calcineurin has a major role in the regulation of cell cycle progression during the early stages of the G1 phase.^11, 66^ Thereafter, several studies have determined the specific molecules regulated by different NFAT members (Figure 4 and Table 2).

The phenotypes of NFAT knockout mice provided strong evidence for the important role of NFAT in cell cycle regulation. Cells from the lymph nodes and spleen of NFAT1^−/−^ mice hyperproliferate in response to different antigen stimulations.^67, 68^ NFAT1^−/−^ lymphocytes have a shorter cell division period upon activation, which is related to an overexpression of cyclins A2, B1, E and F. Moreover, upon activation, NFAT1^−/−^ lymphocytes seem to exit the resting state faster than NFAT1^+/+^ cells. These data suggest that NFAT1 acts as an inhibitor of cell proliferation, regulating very early inducible genes that control the commitment of resting cells to become activated upon stimulation.^69^ NFAT2 deficiency in mice is lethal as a consequence of defects in cardiac valve formation;^8^ nevertheless, in the RAG-deficient complementation system, NFAT2^−/−^ T and B cells showed reduced proliferation when compared with wild-type (WT) mice,^70, 71^ suggesting that, in contrast to NFAT1, NFAT2 might activate genes involved in cell cycle progression.

NFAT1 was shown to directly downregulate cyclin A2 expression by binding to and suppressing the cyclin A2 promoter.^72^ On the other hand, NFAT2 upregulates cyclin A2 expression.^73^ Furthermore, NFAT2 is also involved in upregulation of cyclin D1^74, 75^ and cyclin D3 expression.^76^

The ectopic expression of NFAT1 also inhibits the activity of the human CDK4 promoter. This repression seems to be specific for NFAT1, as no repression was observed with NFAT3 or NFAT4. Apparently, the mechanism by which NFAT1 represses the CDK4 promoter is by competing with E2F family members because the overexpression of NFAT1 inhibited the ability of E2F1–3 to transactivate the CDK4 promoter.^11^ Notably, the NFAT-binding site found in the cyclin A2 promoter lies in close proximity to a putative E2F-binding site.^72^ It would be interesting to evaluate whether the function of NFAT1 in repressing cyclin A2 also occurs by displacing E2F.

Another target for NFAT regulation is the CDK inhibitor p21. p21 is induced by both NFAT1 and NFAT2 in primary mouse keratinocytes, and CsA inhibits the p21 promoter activity associated with Ca^2+^-induced differentiation.^32^ Interestingly, the direct binding of NFAT to the p21 promoter is dispensable and can be replaced by the interaction of NFAT with Sp1/3.^32^

Although the majority of the studies describes NFAT1 as a negative regulator of cell cycle, a few reports show NFAT1 as a positive regulator. Ectopic expression of NFAT1 promotes heterochromatin formation on CDK inhibitor p15 promoter and silencing of p15 expression, resulting in an increased expression of D-type cyclins and their partner kinases CDK4/6, consequently contributing to tumor growth.^77^ The same group has also demonstrated NFAT1 cooperating with STAT3 to promote CDK6 upregulation and pancreatic cancer cell proliferation.^46^ Remarkably, loss of STAT3 expression reversed the NFAT1-dependent stimulation of proliferation, emphasizing how the signaling pathways present when NFAT is activated might convert NFAT from activators to repressors or *vice versa*.^46^

Finally, NFAT also regulates the *c-Myc* gene. It has been shown that NFAT2 induces c-Myc transcriptional activity by binding to a specific element within the c-Myc proximal promoter.^6, 78, 79^ We demonstrated that NFAT1 also regulates the c-Myc promoter.^80^ In addition to this proximal site previously described, NFAT1 directly binds to distal sites of the c-Myc promoter with different affinities. NFAT1 positively regulates the proximal site but acts as a negative regulator once bound to distal elements of the c-Myc promoter. The extent of NFAT1 regulation depends on the cooperation with other partners, such as p300,^80^ again highlighting the importance of protein partners to modulate NFAT function.

It has not been demonstrated whether NFAT3 and NFAT4 directly regulate genes involved in cell cycle progression; however, they do have a role in cell death and survival, as discussed in the following section.

## NFAT and Apoptosis Regulation

**Figure 5:**
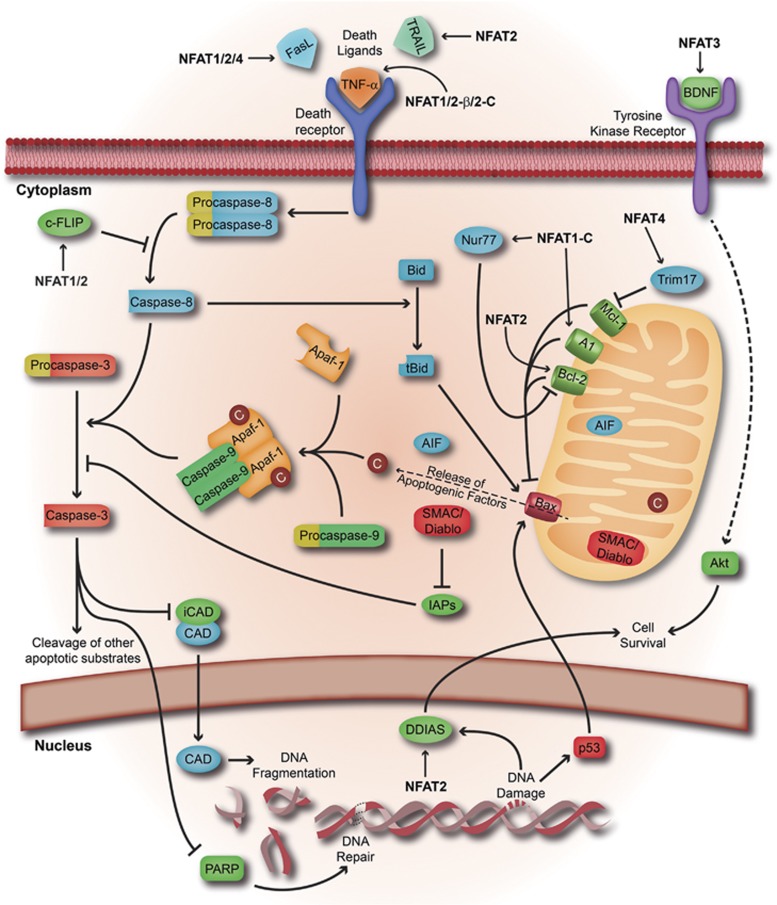
Regulation of the apoptosis signaling pathway by NFAT proteins. Schematic representation of the apoptosis regulation. The extrinsic and intrinsic apoptotic pathways are represented. NFAT proteins control cell survival and cell death by regulating the expression of some apoptotic genes. The negative or/and positive regulations driven by NFAT proteins on these apoptotic-related genes are indicated

The hyperproliferative disorder observed in NFAT1^−/−^ lymphocytes,^68, 69^ as well as the lymphadenopathy and splenomegaly exhibited by double-knockout NFAT1/NFAT4 mice,^81^ has been linked to resistance to cell death. In contrast, whereas peripheral NFAT2^−/−^ T cells show impaired proliferation but no apparent defects in apoptosis,^82^ NFAT2^−/−^ B cells exhibit increased activation-induced cell death compared with WT cells.^83^ Altered proliferation or apoptosis patterns have not been reported in NFAT3^−/−^,^84^ but it was shown that NFAT3 does promote survival in neuronal cells.^85, 86^ The NFAT4 single knockout mouse displays increased apoptosis of both double-positive thymocytes and peripheral T cells.^28, 87^ Likewise, NFAT4 protects pulmonary cells from apoptosis under hypoxia.^88^ A more recent study, however, reports the involvement of NFAT4 with the promotion of cardiomyocyte apoptosis.^89^ Whether specific NFAT4 isoforms or protein partners expressed in different cell types explain the distinct phenotypes induced by NFAT4 remains to be demonstrated. All of these data indicate that each NFAT gene exhibits individual properties, suggesting that the cellular threshold level for each protein and the cell type might determine which set of target genes will be expressed. Next, we discuss the pro- and anti-apoptotic genes regulated by individual NFAT proteins and their roles in cell physiology (Figure 5 and Table 2).

## Anti-apoptotic Genes

### *c-FLIP*

This protein acts as an apoptosis inhibitor through modulation of caspase-8/10 activity.^90^ Recently activated T cells are resistant to FasL-mediated cell death, presumably because of increased expression of anti-apoptotic molecules.^91^ In contrast to naive or long-term activated T cells, short-term activated T cells strongly upregulate the short splice variant of c-FLIP, c-FLIP_s_. The induction of c-FLIP_s_ in T cells is primarily mediated by the NFAT pathway, and blockage of NFAT-mediated c-FLIP_s_ expression rendered T cells sensitive toward FasL-induced cell death.^91^ Interestingly, whereas NFAT1 and NFAT2 proteins bind and transactivate the c-FLIP promoter, NFAT3 is unable to promote c-FLIP expression.^91^

### *A1*

The Fc*ɛ*RI activation-induced survival of mast cells is dependent on the prosurvival protein A1, a member of the anti-apoptotic Bcl-2 family.^92^ A NFAT-binding site was characterized in the A1 promoter, and inhibition of NFAT activation by CsA treatment abrogated the expression of A1 in mast cells.^93^ Furthermore, CA-NFAT1-C but not CA-NFAT2-*β* overexpression increases A1 mRNA levels.^93^ This difference might be explained by the presence of the long TAD-C present in NFAT1-C that is absent in NFAT2-*β*. As previously discussed, the TAD-C domain works as a transactivation domain and can bind NFAT partner proteins important for A1 transactivation.

### *DDIAS*

NFAT2 directly induces the transcription of DDIAS,^94^ an anti-apoptotic protein highly expressed in lung cancers.^95^ A positive correlation between DDIAS and NFAT2 expression was found in human lung tumors, and inhibition of NFAT2 or DDIAS increased cell death in the presence of cisplatin, providing insights into DDIAS-associated chemoresistance via NFAT2 activation.^94^

## Pro-apoptotic Genes

### *FasL*

The resistance to apoptosis observed in T lymphocytes from double-knockout NFAT1/NFAT4 mice could be explained by the remarkably impaired expression of the *FasL* gene in these cells.^81^ Treatment of lymphocytes with CsA inhibits TCR-mediated FasL expression.^96, 97, 98^ Both NFAT1 and NFAT2 proteins bind to two sites within the FasL promoter,^96^ and ectopically expressed NFAT1-C transactivates the FasL promoter in T cells.^97, 99^ The effect of NFAT in the transcription of FasL is at least in part indirect, via Egr3 (early growth response-3). NFAT1 or NFAT4 directly transactivates the Egr*3* promoter, which, in turn, transactivates the FasL promoter.^98^ However, the magnitude of Egr3 induction in activated T cells isolated from single NFAT1^−/−^ or NFAT4^−/−^ mice is normal, suggesting that NFAT1 and NFAT4 could have a compensatory role in the transactivation of the *Egr3* gene and, consequently, on FasL expression.^98^

### *Nur77*

Nur77 mediates negative selection in thymocytes after strong TCR engagement in the thymus.^100^ The Ca^2+^-responsive element in the Nur77 promoter contains two binding sites for MEF2, which cooperates with coactivators to recruit histone acetylases and activates the Nur77 expression.^101^ One of these coactivators might be NFAT1, because NFAT1–MEF2 can cooperatively bind to the Nur77 promoter and induce its expression. Interestingly, this NFAT1–MEF2 cooperation does not depend on NFAT binding to DNA. Moreover, the complex MEF2D–NFAT1 still recruits p300 for the full transcriptional activation of Nur77.^59^ Because MEF2 and p300 interact with both NFAT1-C TAD-N and TAD-C, it becomes evident that splicing of NFAT members is an important step in the regulation of Nur77 and, consequently, in determining the cell fate.

### *TNF-α*

Four putative NFAT-binding sites have been demonstrated in the TNF-*α* promoter.^102, 103, 104, 105^ Although all NFAT members bind to the TNF-*α* promoter,^106^ NFAT1 binds to all the elements, whereas NFAT2 apparently only binds to the distal site.^103, 104, 107, 108, 109^ It was shown that NFAT1-C can induce TNF-*α* transcription more efficiently than NFAT2-*α.*^109^ This difference was mapped to the long TAD-C domain present in the NFAT1-C protein because a hybrid of NFAT2-*α* fused to NFAT1-C TAD-C increases the TNF-*α* promoter transactivation. Furthermore, the NFAT2-C long isoform also transactivates the TNF-*α* promoter less efficiently than the NFAT1-C protein.^109^ These results are in accordance with a previous work, which demonstrated that NFAT2-*α* does not promote cell death of primary T lymphocytes, whereas the NFAT1 and NFAT2 long isoforms do induce apoptosis.^110^ Recently, our group showed that NFAT2-*β* also induces apoptosis through the upregulation of TNF-*α* expression. Similarly to NFAT2-*α*, NFAT2-*β* also lacks the long TAD-C but it has a highly acidic domain in the TAD-N, which confers high capacity to transactivate the TNF-*α* promoter (black square, Figure 3).^111^ Thus, the inability of NFAT2-*α* to induce apoptosis lies in the lack of both the long TAD-C and the acidic domain present in TAD-N.

### *Trim17*

Trim17 is an essential protein for neuronal apoptosis,^112^ which interacts with NFAT3–4 and inhibits their function by decreasing NFAT nuclear localization. NFAT4 binds to a NFAT site in the Trim17 promoter, and overexpression of NFAT4 increases Trim17 expression and aggravates cell death. Accordingly, shRNA against NFAT4 protects neurons from apoptosis and reduces Trim17 mRNA. Conversely, NFAT3 overexpression protects neurons from apoptosis, with no effect in Trim17 expression.^63^ Thus, this study suggests a mechanism that explains the opposite role for NFAT3 and NFAT4 on neuronal apoptosis.

The effect of individual NFAT proteins in apoptosis regulation might rely on their ability to control the expression of both pro- and anti-apoptotic genes at the appropriate stage of activation and differentiation. Different NFAT proteins are specific and prominent regulators of apoptosis and cell cycle genes. It is important to have more studies comparing the function of different members and isoforms in the same model to elucidate the role of each one.

## NFAT and Tumorigenesis

As described in the previous sections, the NFAT family has an important role in the regulation of cell cycle progression, gene expression and apoptosis. All these studies indicate that the Ca^2+^/NFAT signaling pathway is essential to maintain normal cell physiology, and, therefore, the deregulation of this pathway can be associated with malignant cell transformation. In fact, deregulation of calcineurin/NFAT signaling and/or abnormal expression of its components have been reported in solid tumors of mesenchymal and epithelial origin, lymphoma and leukemia.^113^ In this section, we focus on the different roles described for the NFAT members in tumor development.

To investigate the effects of sustained NFAT2 signaling in preadipocyte cells, Neal and Clipstone^114^ reported that the expression of the CA-NFAT2-*α* leads these cells to adopt the well-established hallmarks of cellular transformation, providing direct evidence for the oncogenic potential of NFAT2.^114^ Later, NFAT2 was found to be ectopically expressed and highly activated in pancreatic cancer cells.^6^ Importantly, the proliferation and the anchorage-independent growth of cultured pancreatic cancer cells were significantly attenuated by CsA or siRNA against NFAT2.^6^ Active nuclear NFAT2 was also found in cases of Burkitt lymphoma, diffuse large B-cell lymphoma, aggressive T-cell lymphoma and bladder cancer.^35, 115, 116, 117^ More recently, NFAT2 was also implicated in colon cancer cell invasion and metastasis^118^ and in prostate cancer progression.^119, 120^ Of note, besides all those studies reporting *NFAT2* as an oncogene, it was shown that one of the antitumor effects of arachidonic acid treatment in mammary and pancreatic cancer cell lines was through the induction of NFAT2, which downregulates GLI-1 transcription, resulting in decreased expression of anti-apoptotic genes such as *Bcl-2* and *4-1BB*.^121^ However, it remains to be clarified whether a specific NFAT2 isoform is induced in this context or whether the arachidonic acid modifies the expression of putative NFAT2 partners.

Those phenotypes of oncogenicity induced by CA-NFAT2-*α* were also demonstrated by our group in NIH3T3 fibroblast cells.^64^ Remarkably, we showed that, in contrast to CA-NFAT2-*α*, CA-NFAT1-C was neither able to transform NIH3T3 cells nor to induce tumors in athymic mice. Furthermore, CA-NFAT1-C expression reduced cell growth, the number of *foci* in culture and induced apoptosis of cells transformed with either CA-NFAT2-*α* or H-rasV12 oncogene. Strikingly, when challenged with a chemical carcinogen, the NFAT1^−/−^ mice were more susceptible to tumor development than the WT mice, suggesting an important role for NFAT1 in tumor suppression.^64^ The tumor-suppressor role for the NFAT1-C protein was further mapped to the TAD-C domain.^64, 122^ The antitumor activity of NFAT1 was first proposed in a chondrocyte lineage, based on observations of uncontrolled proliferation of abnormal extra-articular cartilage cells in NFAT1^−/−^ mice.^4^ Recently, it was shown that NFAT1^−/−^ mice also develop spontaneous B-cell lymphomas.^123^ Taken together, these results support an opposite role for NFAT1 and NFAT2 in the regulation of tumorigenesis.

Despite the fact that a cell-intrinsic tumor-suppressor role for NFAT1 has been established during the last decade,^4, 64^ a second role for NFAT1 in tumor progression has been demonstrated. NFAT proteins also promote migration and invasion through *α*6*β*4 integrin signaling.^7^ High levels of NFAT1 and NFAT5 were found in tissue sections from patients with invasive ductal breast carcinoma, where the expression of the *β*4 integrin was restricted to tumor tissue and was absent in normal breast tissue. The signaling through *α*6*β*4 integrin activates NFAT1, NFAT4 and NFAT5.^7^ Consistent with these data, the Akt-dependent NFAT1 ubiquitination and its subsequent degradation proteasome resulted in impaired migratory and invasive potential of the breast cancer cell lines.^124, 125^ NFAT1 also regulates the invasion of glioblastoma multiforme cells^126^ and metastasis of mammary cancer cells.^127^ NFAT1 was also found ectopically expressed in the nucleus of pancreatic tumors. It was shown that an inflammatory microenvironment induces NFAT1 activity, which in turn contributes to tumor growth.^46, 77^ Furthermore, a crucial role for NFAT1 in initiation and promotion of inflammation-associated colorectal tumors was reported.^128^ In addition, some recent studies have implicated NFAT factors with the dedifferentiation of cancer cells. NFAT1 promotes the dedifferentiation of melanoma cells,^129^ whereas NFAT3 induces acinar to ductal dedifferentiation and pancreatic cancer initiation.^130^ Moreover, in pancreatic cancer, NFAT2 drives epithelial–mesenchymal transition and seems to be responsible for the appearance of stem-cell features.^131^ These studies demonstrate that NFAT factors control the plasticity of some cancer cells and might be involved in the maintenance of cancer stem cells. However, the mechanisms linking cancer cell dedifferentiation driven by NFAT and stemness remain to be demonstrated.

These findings also show that changes in the transducing pathways are the key elements to pathogenesis of cancer. NFAT proteins may be overexpressed or inactivated, becoming dangerous to the cell. During tumor development, alterations in NFAT regulation may occur, leading to the expression of different protein levels and/or isoforms as well as alterations in the expression of partner proteins. Consequently, NFAT proteins can contribute to distinct aspects of the tumorigenic phenotype, including the initial deregulation of cellular growth control, the recruitment of an adequate blood supply and the regulation of tumor metastasis. Indeed, the importance of NFAT as a cancer therapeutic target has been raised in the last few years (reviewed by Qin *et al.*^132^ and Metzelder *et al.*^133^). However, the subsets of genes that are regulated by NFAT proteins during the different stages of tumor progression remain to be elucidated.

## Concluding Remarks

NFAT proteins have been described in many cell types and regulate genes involved in cell cycle progression, cell differentiation and apoptosis. The role of NFAT proteins in these pathways reveals a fundamental function for this family in normal cell physiology. Although NFAT members share conserved domains, the TADs are highly variable regions, which can have a critical role in NFAT function because they can contribute to non-redundant functions. In fact, it has been shown that the TADs are sites of interaction between distinct NFAT family members and several partners. The differential levels and splicing variants of NFAT proteins and the cell type in which they are being expressed can determine whether the cell will live, proliferate or die. Beyond that, the deregulation of NFAT signaling is clearly involved in tumorigenesis, where NFAT2 acts as an oncogene, whereas NFAT1 acts as an oncogene or a tumor-suppressor gene depending on the cells and tissue types that were evaluated. The detailed identification of the isoforms expressed in different tumors will elucidate whether different NFAT isoforms have distinct roles during the tumorigenesis process.

## Figures and Tables

**Table 1:** NFAT partners in transcription

**Partner**	**NFAT member**	**Technique**^a^	**Region of interaction on NFAT**	**Effect on transcription**	**Gene regulated**	**Reference**
AP-1 (c-Fos/c-Jun)	NFAT1; 2; 3; 4	A; B; E	DBD	Activation	Several cytokines	^41^
			TAD-C		IL-2	^44^
CBP/p300	NFAT1-C	A; B	TAD-N	Activation	IL-2; TNF-*α*	^54, 55, 56^
	NFAT2-*β*		NHR			
	NFAT3-A		TAD-C			
C/EBP	NFAT1-C	A	NHR-DBD	Activation	PPAR-γ IGF-2	^43^
	NFAT3-A					
c-Maf	NFAT1	C	ND	Activation	IL-4	^29^
c-rel (NFκB)	NFAT2-*α*	A	ND	Activation	CD40L	^35^
EGR1; EGR4	NFAT1; 2	A; B	ND	Activation	IL-2; TNF-*α*	^31^
ER*α*/ER*β*	NFAT3-A	B; C	TAD-N	Inhibition	IL-2	^57, 58^
			NHR			
			TAD-C			
Foxp3	NFAT1-C	A; E	DBD	Inhibition	IL-2; IL-4; IFN-γ	^50, 51^
GATA-2	NFAT2	A	ND	ND	ND	^36^
GATA-4	NFAT3	A; C	ND	Activation	BNP	^37^
GATA-3	NFAT1-C	A	DBD	Activation	IL-5	^38^
	NFAT2-*α*					
ICER	NFAT1-C	B	DBD	Inhibition	IL-2; TNF-*α*	^52^
ICSBP	NFAT2-*α*	A; B	NHR-DBD	Activation	IL-12	^42^
IRF2BP2	NFAT1-C	B; C	TAD-C	Inhibition	IL-2; IL-4; TNF-*α*	^62^
IRF4	NFAT1	A; B	ND	Activation	IL-4	^30^
MEF2D	NFAT1-C	A	TAD-C	Activation	Nur77	^59^
Mrj	NFAT4-x1	A; C	DBD	Inhibition	TNF-*α*	^49^
NIP45	NFAT1-C	A; C	DBD	Activation	IL-4	^39^
PPAR-γ	NFAT2-*α*	A; B	DBD	Inhibition	IL-4; IL-2	^47, 48^
PU.1	NFAT2	A	ND	Activation	Cathepsin K	^33, 34^
Rack-1	NFAT2	A; C	DBD	Inhibition	3x NFAT	^53^
Raf-1	NFAT4	A	ND	Activation	CXCR5	^45^
RAR/RXR	NFAT1	B	DBD	Activation		
	NFAT2		ND	Inhibition of NFAT1-dependent activation	CCR9	^134^
Sp1/Sp3	NFAT1; 2	A	ND	Activation	p21	^32^
Sox10	NFAT3-A	A; D	DBD	Activation	Krox20	^40^
STAT3	NFAT1	A	ND	Activation	CDK6	^46^
Trim17	NFAT3	C	TAD-C	Inhibition	BDNF	^63^
	NFAT4					

Abbreviation: ND, non determined.

a Co-immune, A; GST pull-down, B; two hybrid, C; mass spectrometry, D; X-ray analysis, E.

**Table 2:** Genes regulated by NFAT

**Function**	**Gene**	**NFAT member or isoform**	**Effect on transcription regulation**	**Reference**
Cell cycle	CDK4	NFAT1-C	Downregulation	^11^
		NFAT3 and 4	No effect	
	CDK6	NFAT1	Upregulation	^46^
	c-Myc	NFAT2-*α*	Upregulation	^6^
		NFAT1-C	Down/upregulation	^80^
	Cyclin A2	NFAT2-*α*	Upregulation	^73^
		NFAT1-C	Downregulation	^72^
	Cyclin D1	NFAT2-*α*	Upregulation	^74^
		NFAT1 and 3	No effect	
	Cyclin D3	NFAT2	Upregulation	^76^
	p15	NFAT1	Downregulation	^77^
	p21	NFAT1 and 2	Upregulation	^32^
		NFAT3 and 4	No effect	
Anti-apoptotic	A1	NFAT1-C	Upregulation	^93^
		NFAT2-*β*	No effect	
	Bcl-2	NFAT2	Upregulation	^135, 136^
	BDNF	NFAT3	Upregulation	^86^
		NFAT4	No effect	
	DDIAS	NFAT2	Upregulation	^94^
Pro-apoptotic	c-FLIP	NFAT1 and 2	Upregulation	^91^
		NFAT3	No effect	
	FasL	NFAT1, 2 and 4	Upregulation	^96, 97^
	Nur77	NFAT1-C	Upregulation	^59, 101^
	TNF-*α*	NFAT1, 2-*β* and 2-C	Upregulation	^103, 107, 108, 111^
		NFAT2-*α*	No effect	
	TRAIL	NFAT2	Upregulation	^137^
	Trim17	NFAT3	No effect	^112^
		NFAT4	Upregulation	

## Abbreviations

NFAT: nuclear factor of activated T cells
CA-NFAT: constitutively active NFAT
NHR: NFAT-homology region
DBD: DNA-binding domain
TAD: transactivation domain
AAD: acidic activation domain
AP-1: activator protein-1
IL: interleukin
ER: estrogen receptor
MEF: myocyte enhancer factor
IRF2BP2: interferon regulatory factor 2-binding protein 2
CBP: CREB-binding protein
TNF: tumor necrosis factor
c-FLIP: cellular FLICE-like inhibitory protein
STAT: signal transducer and activator of transcription
DDIAS: DNA damage-induced apoptosis suppressor
CsA: Cyclosporin A
CDK: cyclin-dependent kinase
CKI: CDK inhibitor
SRR: serine-rich region
SP: serine-proline motif
NLS: nuclear localization signal
NES: nuclear export sequence
WT: wild type
